# Ground reference data for sugarcane biomass estimation in São Paulo state, Brazil

**DOI:** 10.1038/sdata.2018.150

**Published:** 2018-08-07

**Authors:** Ramses A. Molijn, Lorenzo Iannini, Jansle Vieira Rocha, Ramon F. Hanssen

**Affiliations:** 1 Geoscience and Remote Sensing, Delft University of Technology, Delft 2628CN, The Netherlands; 2 FEAGRI, Unicamp, Campinas 13083-875, Brazil

**Keywords:** Agriculture, Ecological modelling, Agroecology

## Abstract

In order to make effective decisions on sustainable development, it is essential for sugarcane-producing countries to take into account sugarcane acreage and sugarcane production dynamics. The availability of sugarcane biophysical data along the growth season is key to an effective mapping of such dynamics, especially to tune agronomic models and to cross-validate indirect satellite measurements. Here, we introduce a dataset comprising 3,500 sugarcane observations collected from October 2014 until October 2015 at four fields in the São Paulo state (Brazil). The campaign included both non-destructive measurements of plant biometrics and destructive biomass weighing procedures. The acquisition plan was designed to maximize cost-effectiveness and minimize field-invasiveness, hence the non-destructive measurements outnumber the destructive ones. To compensate for such imbalance, a method to convert the measured biometrics into biomass estimates, based on the empirical adjustment of allometric models, is proposed. In addition, the paper addresses the precisions associated to the ground measurements and derived metrics. The presented growth dynamics and associated precisions can be adopted when designing new sugarcane measurement campaigns.

## Background & Summary

**Figure 1:**
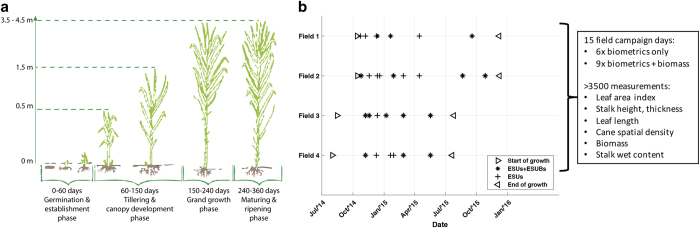
Sugarcane phenological development and data acquisition scheme. (**a**) sugarcane growth stages with indicative heights and plant geometries, adapted and modified from ref. 27; (**b**) ground reference data acquisition scheme, accompanied by a summary of the data collection.

Sugarcane is the number one crop worldwide in terms of production quantity. It provides for more than 40% of the car fuel in the largest sugarcane producing nation, Brazil. São Paulo state is responsible for more than half of the national bio-ethanol production from sugarcane, which expanded more than 100%, 2 million hectares, over the last 15 years. The crop is usually grown for five to seven years from the same ratoon^1^, i.e. the plant sprouts from the same root system after each harvest event. The cycle of sugarcane can vary from 8 to 24 months depending on the cultivars and planting date, with the 12 month and 18 month types dominating the Brazilian plantations^2^. The growth stages after planting consist of the germination phase, when the first leafs and stems that are part of the root system emerge, the tillering and canopy development phase, indicatively from the second to the fifth month when secondary stems arise above ground, the grand growth phase, between the fifth and eighth month, and finally the maturing and ripening phase until harvest, see the left part of Fig. 1. The sucrose content, from which the bio-ethanol and sugar is produced, is concentrated in the stem, also referred to as stalk, of the sugarcane. Sugarcane yield is commonly measured in tons cane per hectare (TCH)^3–6^ and ranges up to 160 TCH^6,7^.

In order to improve the understanding of the plant itself and of the related impacts, several sugarcane measurement campaigns have been conducted in Brazil for a wide range of studies, most notably including biological analysis of plant processes^6,8–13^, agronomic analysis on the effects of different fertilization and soil practices^4,5,14–16^ and bio-physical parameter extraction from space-based remote sensing signals^7,10,17–22^. Due to the plant morphology and its growing environment, sugarcane measurements are labour-intensive and easily affected by significant errors. Quantifying uncertainty metrics in the collected and processed measures is extremely valuable in data modeling and data integration frameworks. However, so far, only a few contributions^5,6,19,23^ explicitly accounted for the quality of their reference ground data.

With such premise, we planned a ground surveying and data elaboration strategy that could entail an in-depth uncertainty analysis. The purpose for conducting the measurement campaign was to investigate the sensitivity of optical and radar satellite imagery to sugarcane biophysical features along the whole growth cycle. Nevertheless, the produced dataset can valuably fit a variety of other sugarcane studies. The biophysical parameters of interest include: cane height, cane density, leaf area index (LAI), cane biomass and leaves biomass. The measurements were carried out at four fields in the São Paulo state, selected for their heterogenous conditions in terms of crop health and ratoon cycle. The fields were surveyed multiple times at different development stages. The campaign addressed two additional needs: that of minimizing the measurements' effects on the remote sensing signals and that of maximizing efficiency in terms of costs. Both demands led to the limitation of the destructive biomass procedures. As a result, the majority of the surveying locations, denominated Elementary Sampling Units (ESU), comprise only biometric measurements. The biomass weighting operations were then added to a selected number of locations, hence recalled Elementary Sampling Units with Biomass (ESUB). Consequently, a method to retrieve biomass from non-destructive biometric measurements at ESUs was adopted. In this method, the destructive measurements at ESUBs are used to calibrate the allometric models and to cross-validate their outcome. The correlation coefficient between the directly measured and estimated biomass values is 0.89. The selection of the surveying locations and the data collection and processing procedures will be explained in the sec:Methods section.

The uncertainty analyses (expressed in precisions, see the sec:Technical-Validation section) are based on spatially-intensive repetitive measurements and on the error propagation laws applied to the biomass equations. For cane biomass, it was expected and shown that the instrument operation precision is smaller than the precision associated to the local plant variations. The precision related to model fitting and data interpolation procedures contributes significantly to the total precision, but it reduces over time. The relative precision on indirect biomass measures is hence lower for mature cane. As expected, the leaf biomass precisions dominate the total precision at an early stage but are quickly taken over by the stalk-related precisions.

Finally, based on the findings, we like to make some recommendations on setting up novel ground measurement campaigns. First of all, planning the ESU and ESUB locations for taking ground measurements requires thorough preparation beforehand, predominantly counting for the costs of the repetitive measurements needed to derive the associated precisions. Based on high-resolution optical images one should select those ESU locations for which the difference in biophysical parameter is expected to be statistically significant within the fields of interest. The proposed techniques as explained in this paper can be used accordingly.

## Methods

**Figure 2:**
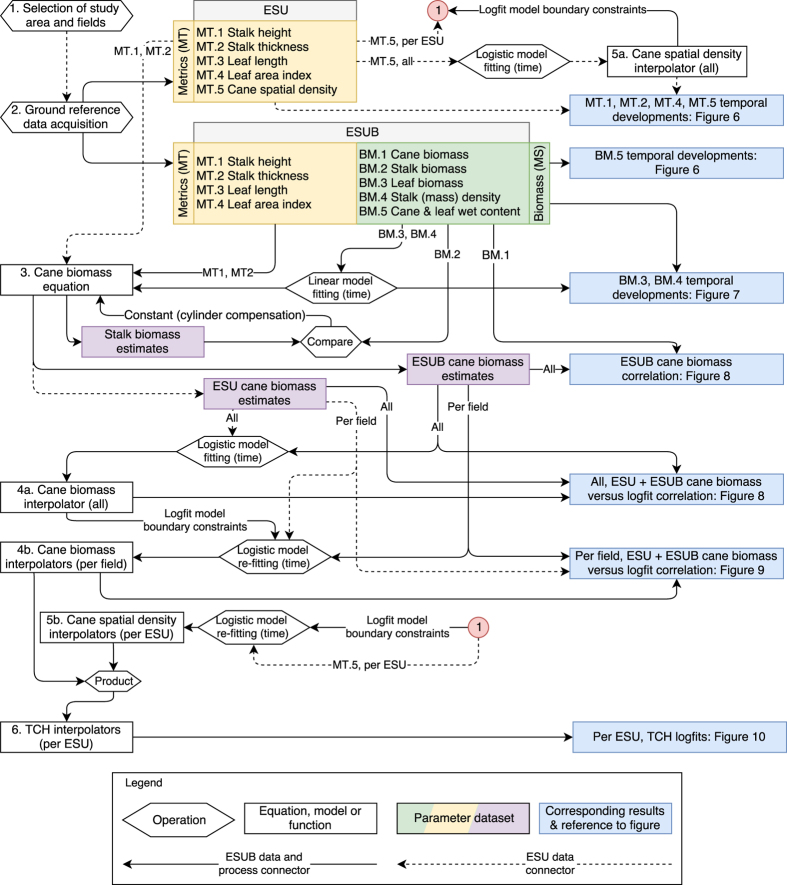
Workflow indicating the operations, relations between the methods and references to the corresponding resulting figures.

The relationships between the methods and the corresponding figures are schematized in Fig. 2. Details on the practices and additional information on the linkages will be given in the following sections with references to the numbers in the figure.

### Selection of study area and fields

**Figure 3:**
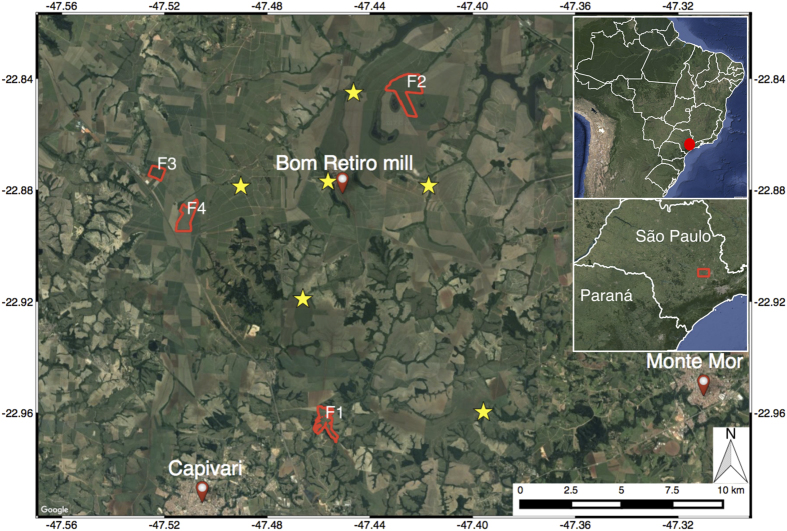
Study area, indicating the four fields of interest with red polygons and the weather stations with yellow stars.

The sugarcane fields of interest were proposed by the fields' owner, one of the largest sugarcane producers and energy companies in Brazil, based on their availability, accessibility and quality. The fields are located in an area densely covered by sugarcane plantations, spread around the Bom Retiro sugarcane mill, twenty kilometers from Piracicaba, São Paulo state, Brazil, see Fig. 3. They are mostly flat and contain inter-field and intra-field roads of two to ten meters wide. The fields' characteristics, see Table 1, show that there are differences in ratoon cycles, area and start and end of growth. All fields are situated on clay grounds.

**Figure 4:**
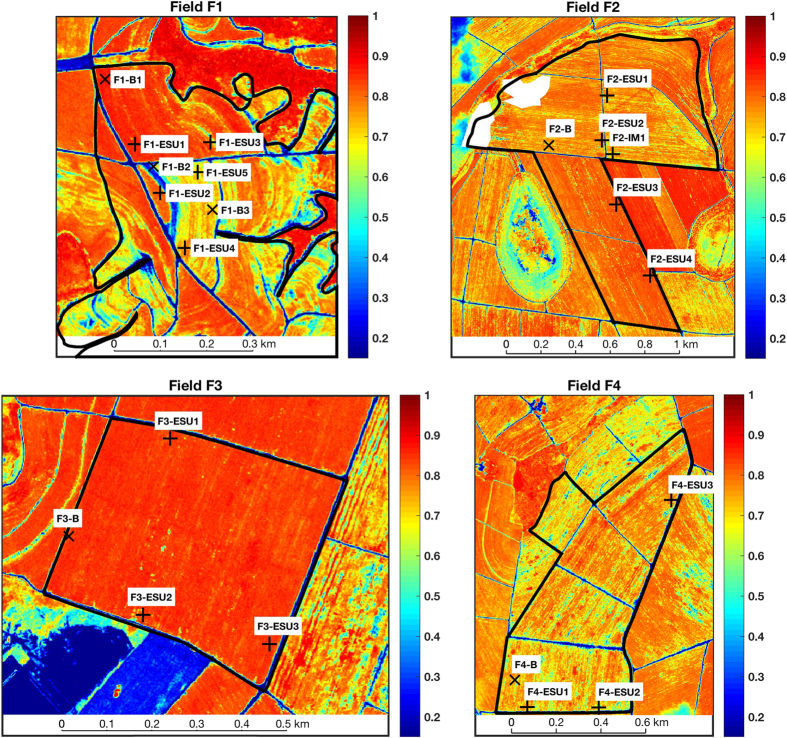
Delineation of the four studied sugarcane fields and ESU and ESUB (**b**) locations. The background map shows the NDVI from WorldView-2 on January 25, 2015. The plus signs (+) and crosses (x) show, respectively, the location of the elementary sampling units (ESUs) and elementary sampling units for biomass (ESUBs). The white patches in field F2 are masked clouds.

In order to select the locations to measure, we visually analyzed optical images from Landsat-8 from previous years and identified consistent anomalies in NDVI values, which are indicative for sugarcane biomass differences^7,24^. Subsequently, we split the locations into elementary sampling units (ESUs) for the regular biophysical parameter measurements and elementary sampling units for biomass (ESUBs). Three to five ESUs and one or two ESUBs per field were selected, see Fig. 4. These ESUs and ESUBs were visited multiple times during one growth season, see Fig. 1, using a GPS location device featuring approximately five meters location accuracy.

### Ground reference data acquisition

**Figure 5:**
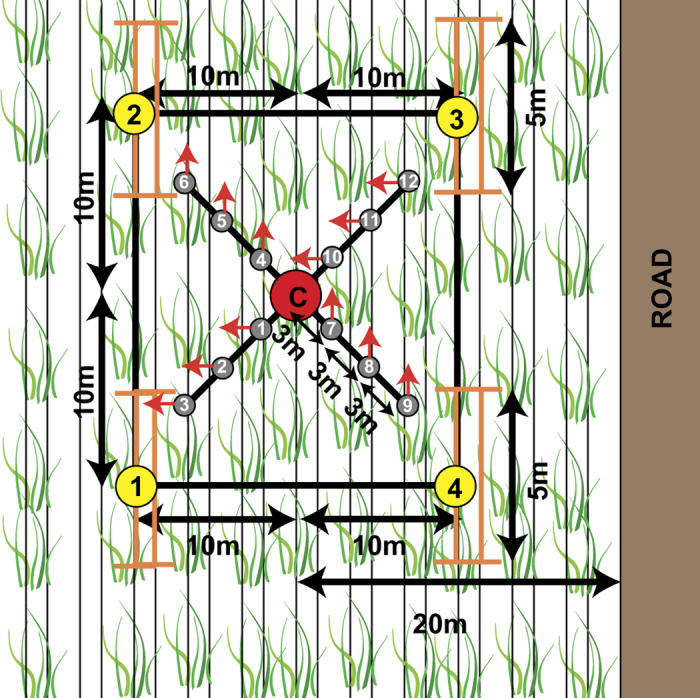
Schematic overview of the ESU measurements. The LAI measurements are taken at 12 different locations (grey circles) in four different directions (red arrows) around the center point (C), which lies 20 meters from the road. The biometric measurements are taken at points 1 to 4 (yellow circles), including the number of plants and canes along two rows spanning 5 meters (orange lines).

**Figure 6:**
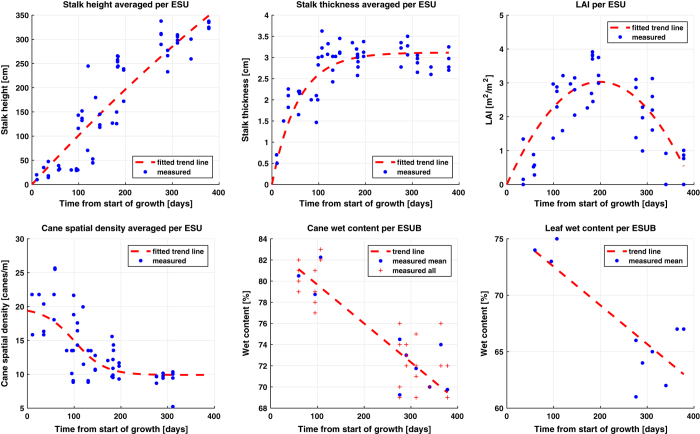
Temporal developments of measured stalk height and thickness, (actual) LAI and cane spatial spatial density, averaged per ESU, and cane wet content and leaf wet content, averaged per ESUB. The fitted trend lines are indicative and only for illustrative purposes.

The measurements in each ESU were taken systematically following a strict procedure. Fig. 5 illustrates schematically the methodology for taking the LAI, biometrics and biomass measurements, as explained in the following subsections. Distinction is made between plant, cane, stalk and leafs, whereby the plant is the collection of canes emerging from the same root system and cane is the integral of the stalk and leafs. The temporal developments of the measured parameters are illustrated in Fig. 6. The different behaviors in time, as indicated by the illustrative trend lines, are addressed at each parameter description.

### Biometrics

At all four corner points the following biometric measurements were taken:

- stalk heights of highest canes. The stalk height is here defined as the length from the stalk at ground level to the crown from which the upper leafs emerge,
- stalk thicknesses of the same canes, taken at the stalk on ground level,
- longest leaf lengths of the same canes,
- number of canes and plants per meter by counting the number of canes and plants over five meters distance for two adjacent rows, as a measure for cane spatial density.

The highest canes were selected by eye. As will be explained in subsequent sections, the variations in modeled intra-field biomass values are dictated by the measured cane spatial density at the ESUs. Hence, the selection of the highest canes mainly serves to establish the general biomass curve and subsequently the field-wide cane biomass interpolators (see Section subsec:Cane-biomass-interpolators). This addresses the potential issue that the selection of the measured (highest) canes may not be representative for estimating the cane biomass per unit area (expressed in tons cane per hectare-TCH) at the measurement locations. The dataset also includes the measurements of smaller canes at each measurement location. The smallest canes that are unrepresentative for the sugarcanes at the measurement location were not taken into account, for example sprouting canes amongst mature canes. These, however, are not used in any further modeling or estimation.

In addition to the biometrics, photos embedded with the geo-location were taken for location verification, for checking the leaf angle distribution and for future referencing. The indicative trend lines come from a fitted linear function for height and a logistic function, showing stabilization after a certain moment in time, for stalk thickness (increasing in time). For cane spatial density, a logistic function as a function of time was fitted as well, which is used in a later stage to constrain the re-fitted functions at ESU-level, see *“4a”* and *“4b”* in Fig. 2. This will be explained later in this section in more detail. The shapes of the resulting curves are in line with the reported trends in^11,13,19^, although the cane spatial density in these studies first shows a slight increase in the first 150 days before the decrease. For the cane height and stalk thickness the functions are constrained to pass through the zero origin.

### Leaf Area Index (LAI)

For LAI measurements, a LICOR LAI-2000 Plant Canopy Analyzer was used with the default calibration values as reported in the LAI manual^25^. The manual and^26^ were consulted for setting up the following procedure. During the first growth stages of the sugarcane, until approximately two meters stalk height, the LAI measurements are taken from the middle of the ESU in four different directions with one above-canopy measurement followed by three below-canopy measurements; two times in cross-row direction and two times in along-row direction, see Fig. 5. After this point in time, the measurements showed negligible differences between the directions and we assumed the canopy coverage became planar isotropic; the coverage of the leafs is uniform in both horizontal directions. Consequently, the LAI measurements are taken with one above-canopy measurement followed by nine below-canopy, all of them within ten meters in row direction divided over three rows. As for the precise placement of the sensor for all below-canopy measurements, the distance to the plants was taken into account, i.e. per set of three measurements one measurement was taken adjacent to the cane, one at one third of the row spacing and the last at two third of the row spacing. LAI measurements in the direction of the road were avoided to minimize the possible road effects. Also the climatic conditions were taken into account by retaking above canopy measurements when clouds were moving. The resulting measured LAI can also be referred to as effective LAI since it is not corrected for the leaf clumping effect.

The correction from effective LAI to actual LAI was carried out as follows. Along two meters and for two rows wide (1.5 + 0.9 meters) the LAI was measured in four different directions and three measurements per direction with spacing of one meter (instead of three meters as for the ESU measurements). Within this area, around the center point all plants were cut and the green leafs were stripped from the canes, which were then placed on a white sheet. Through supervised classification with standard image analysis software, the area of the green leafs was divided by the total area, i.e. 2.4 by 2 meters. The average of the differences between the measured and observed LAI taken at two dates was subtracted from the LAI measurements. The correction results in a few LAI values to become slightly below zero.

The trend line for actual LAI in Fig. 5 is a second order polynomial fit, which is supported by results presented in ref. 11,13,19,23, and is forced through zero origin. The few LAI values smaller than zero are plotted as zero.

### Biomass

In order to minimize destructive measurements at the ESUâ€™s, which would bias the remote sensing signals, biomass ESUâ€™s (ESUBâ€™s) were selected. Four whole plants (containing generally between ten and twenty canes) were cut at ground level and the number of canes per plant was counted. The stalk heights, stalk thicknesses and leaf lengths were measured per plant similarly as described above. The mass of the entire plant was measured separately and by dividing by the number of canes its average and variation in cane weight was obtained. This will be referred to in the sec:Technical-Validation section. After the weighing, per plant two representative canes were selected and combined, creating a sample of eight canes of the four plants. Leafs were then separated from the stalks and weighed separately, giving the leaf biomass per cane. The mass of these stalks divided by the volume, approximated by the cylindrical volume as a function of the measured stalk thickness and height (see Equation 1), gives the stalk (mass) density. Note that the minor compensation for the tapered geometry of the cylinder, referred to the scaling factor *C* in the equation, is not yet taken into account in Fig. 6. The remaining canes of the fours plants, the stripped leafs and stalks were separately ground into centimeter-sized pieces, a sample was taken, weighed and dried in the oven at 65 C for 72 h. The complement of the ratio between the mass of the dried and the mass of the wet samples gives the wet matter content, i.e. computed individually for the leafs, stalks and four different plants.

The cane wet content and leaf wet biomass show a decrease over time, see Fig. 6, which was assumed to be linear over time (despite the time gap). The general observed tendency that cane and leafs become drier is supported by^11,19^. The former shows similar water content values for stalks, though slightly higher and more constant during the first 100 days. These fitted linear functions are included only for indicative purposes.

The only measured parameters for which functions are fitted to be used in the biomass equation, see Equation 1, are stalk (mass) density and leaf biomass. This operation is indicated in Fig. 2 by *“Linear model fitting (time)”* with *“BM.3”* and *“BM.4”* as inputs. Stalk density shows a linear increasing trend, which can be attributed to the increase in sucrose content as reported by^6,8,24^, which has higher mass density than water (approximately 1.6 g/cm3 and 1.0 g/cm3, respectively).

**Figure 7:**
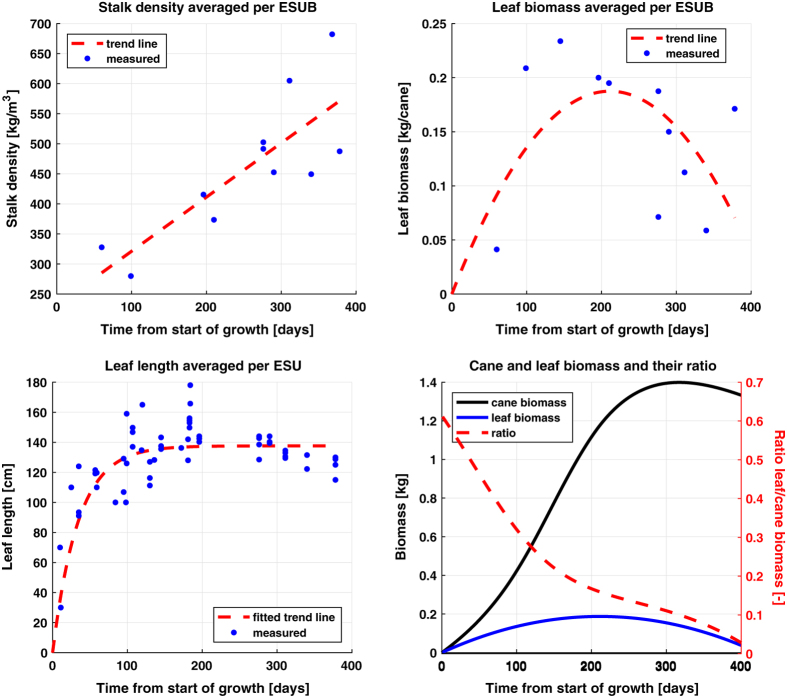
Temporal developments of stalk (mass) density ($\rho_{S}$), leaf biomass per cane ($BM_{L}$), averaged per ESUB, leaf length, averaged per ESU, and cane and leaf biomass and their ratio. The fitted trend lines for $BM_{L}$ and $\rho_{S}$ are used in the biomass equation.

**Figure 8:**
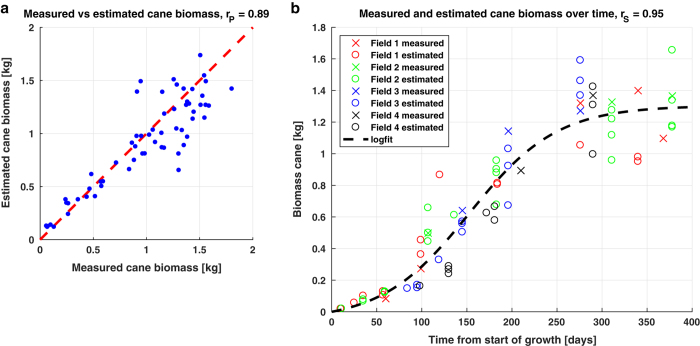
Performance of biomass estimation and performance of modeled growth curve. (**a**) relationship between estimated cane measured and measured cane biomass, accompanied by the (Pearson's) correlation ($r_{P}$); (**b**) general biomass growth curve showing the measured and estimated biomass over time from start of growth from all fields, accompanied by the fitted logistic function and the Spearman's correlation ($r_{S}$).

Similar to LAI, a second order polynomial fit was selected as the trend line for leaf biomass per cane, see Fig. 7. This is based on expected correlation with LAI and findings in the above mentioned LAI-related literature, in particular^11,19^. Due to the low number of measurements, the precision of the function fit is non-optimal. To put the importance of leaf biomass into perspective, we added the approximated development of the ratio of leaf biomass over total cane biomass in Fig. 7. It shows that leaf biomass is dominant to the cane biomass during the first 50 days, after which it rapidly drops to less than 20% of the cane biomass after 200 days and decreases to 5% at the end of the cycle. Hence, the precision of the leaf biomass function has more impact at early state than at later states. The validity of the function is supported however by Fig. 8, showing an acceptable correlation of 0.89 between estimated cane biomass (i.e. as a result from the biomass equation) and measured cane biomass. Acceptable agreement is also found for individual estimations during early state.

### Intensive measurements

In order to assess the reliability of the field measurements, three types of intensive measurements were carried out at three different dates. The details on how these precisions were measured, what their values are and how they are used for further analysis can be found in the sec:Technical-Validation section.

### Precipitation

Over the course of the sugarcane growth cycles, seven weather stations acquired the daily cumulative precipitation, see Fig. 3. The rate of agreement between the weather stations of detecting precipitation was 0.96, which leads to our assumption that all fields are subject to the same condition of precipitation.

### Cane biomass equation

In order to estimate the biomass at ESU we propose a biomass estimation equation, which is based on the sum of stalk biomass (the biomass of the main stem itself) and leaf biomass:

(1)BMC=BMS+BML=((πD)24⋅H⋅ρS(t)⋅F)+BML(t)

*BM*_*C*_ and *BM*_*S*_ are respectively the cane biomass and stalk biomass, *BM*_*L*_ is the biomass leaf per cane, all in kilogram, *D* is the sugarcane stalk diameter in meter, *H* is the sugarcane stalk height in meter, $\rho_{S}$ is the stalk (mass) density in kg/m3 as a function of time, and *F*. The latter, equal to 0.977, is assumed to be a scaling factor that is constant in time and compensates for the stalk as a tapered cylinder. This operation is indicated as *“Compare”* in Fig. 2, it is computed by dividing the ESUB-measured stalk biomass by the estimated stalk biomass from the biophysical parameters not corrected by the constant.

When applying Equation 1 with measured biophysical parameters at the ESUBs, the resulting estimated cane biomass is (linearly) related to the measured biomass with a (Pearson's) correlation of 0.89, see the left part of Fig. 8. The biomass growth curve on the right shows the fitted logistic function in time with the measured and estimated biomass. Since this curve is monotonic but not linear, we express the goodness of fit between the function and the (measured and estimated) biomass through the Spearman's correlation, equal to 0.95.

### Cane biomass interpolators

Since the times of remote sensing image acquisitions rarely coincide with the times of ground measurements, temporal interpolation between field measurements is required. However, the temporal frequency of measurements for each individual field is not sufficient to do this directly for each individual field due to extrapolation and measurement precisions. As such, we combine the biomass measurements (from ESUBs) and biomass estimations (from ESUs) from all fields and create a biomass interpolator as a function of time from start of growth. A logistic function is selected in order to force the sigmoid curve, which is typical for the behavior of growth over time^3–5,21,23^ and was also applied to stalk thickness and cane spatial density:

(2)f(t)=Δy1+e−k(t−t0)

The coefficients consist of Δy, the limiting value defining the horizontal asymptote, *k*, the growth rate of the curve, and *t0*, the midpoint of the sigmoid. The model coefficients are estimated by the non-linear least squares method, without a-priori confidence bounds, using the measured and estimated biomass values, averaged per ESUB and ESU respectively, as input data and the function forced through zero origin. This is indicated in Fig. 2 by operation *“Logistic model fitting (time)”* based on the dataset containing all *“ESU cane biomass estimates”* and all *“ESUB cane biomass estimates”*, resulting in *“4a”*. The result is considered as the general biomass growth curve, see the right part of Fig. 8.

**Figure 9:**
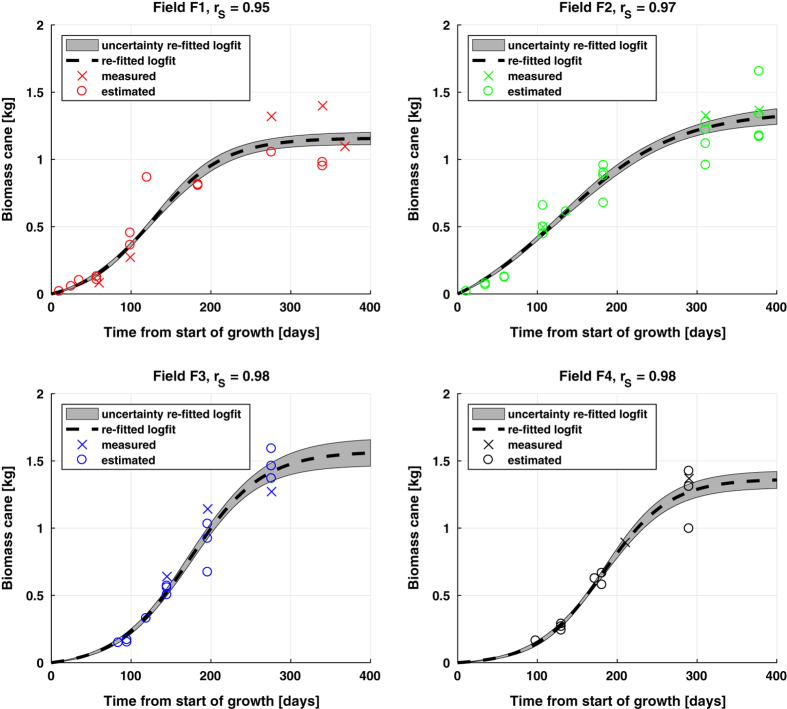
Measured and estimated cane biomass over time from start of growth and the re-fitted logistic fits, per ESU, accompanied by the Spearman's correlation ($r_{S}$) and the associated uncertainty bars (one standard deviation).

Subsequently, for each individual field new logistic functions are re-fitted, see Fig. 9. This re-fitting is constrained by using the estimated coefficients and their 95% confidence bounds from the general biomass growth function, respectively as initial values for the coefficients and as lower and upper bounds on the coefficients to be fitted. The latter bounds were expanded by 10% to allow more flexibility. This is indicated in Fig. 2 as operation *“Logistic model re-fitting (time)”* based on the dataset containing *“ESU cane biomass estimates”* per field and *“ESUB cane biomass estimates”* per field, resulting in *“4b”*. The resulting Spearman's correlations are all equal to or higher than 0.95, which is an improvement with respect to the general biomass growth curve of Fig. 7. The uncertainty bars (one s.d.) are computed with the methodology as depicted in the sec:Technical-Validation section.

### Cane spatial density interpolators

For the cane spatial density, we apply the same logic as for the cane biomass; utilizing the boundary conditions of the general logistic fit, indicated as *“5a. Cane spatial density interpolator (all)”* in Fig. 2, illustrated as the trend line in Fig. 6, to re-fit logistic functions per ESU (i.e. not per field), indicated as *“5b. Cane spatial density interpolator (per ESU)”.*

### TCH interpolators

Finally, in order to obtain the cane biomass per unit area we compute the tons cane per hectare (TCH):

(3)TCH=BMC⋅C(t)⋅10/S

**Figure 10:**
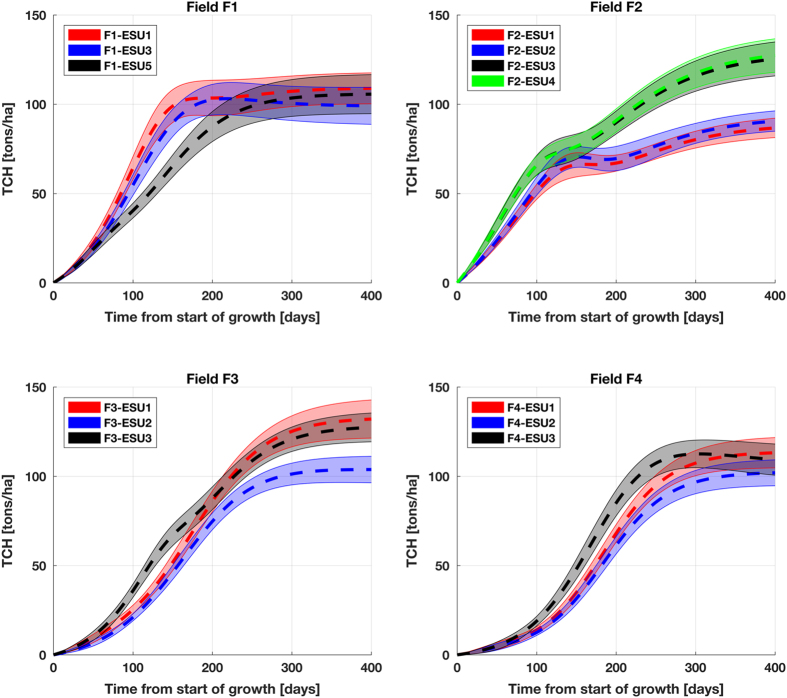
Estimated average TCH over time of start of growth per ESU, along with the associated uncertainty bars (one standard deviation).

$BM_{C}$ is the cane biomass in kg, C(t) is the measured cane spatial density in canes/m2 as a function of time and *S* is the correction factor for spacing between the rows of canes equal to 1.2 (i.e. the average of 0.9 and 1.5). The multiplication by 10 converts kg/m2 to tons/hectare. Multiplying the field-dependent *BM*_*C*_ with the ESU-dependent cane spatial density functions results in the TCH graph, see Fig. 10. Hence, we assume that the sugarcane plant growth within each field is constant and the cane spatial density varies per ESU, with the latter consequently dictating the intra-field differences from the ground reference data. These steps are indicated in Fig. 2 by operation *“Product”* based on *“Cane biomass interpolators (per field)”* and *“Cane spatial density interpolators (per ESU)”*. In the sec:Technical-Validation section the methodology for computing the one s.d. uncertainty bars, as depicted in the figure, is described.

### Code availability

For pre-processing, visualizing and analyzing the presented data, MATLAB scripts are written, which are available upon request.

## Data Records

The ground measurements, published for public use, can be found on the 4TU Centre for Research Data repository, see (Data Citation 1). The dataset contains readme files, which can be consulted for explanations on the location of the data records and supplementary material.

## Technical Validation

For the technical validation of the measured and estimated parameters, three sets of uncertainty metrics are presented. The first set contains the absolute precisions of the measured parameters in the fields. The second set contains the precisions of the computed cane biomass parameter, based on error propagation laws applied on the biomass equation. The third set contains the precisions of the computed TCH parameter, which gives an enhanced view on the reliability of the TCH estimates and the significance of the intra-field differences.

### Absolute precisions of field-acquired parameters

For this, we distinguish between three precision metrics:

- Instrument operation precision: dispersion of the measurement values when measuring the same parameter with the same instrument of the same plant. This precision is estimated through repeatedly measuring the same parameters of the same plant.
- Idealization precision: dispersion of the measurement values when measuring the same parameter with the same instrument at the same intended location but by chance another (neighboring) plant or cane was selected. The diameter of the area in which these plants were selected was five meters, approximately the accuracy of the GPS receiver. As a result, it was simulated that the measurements were taken at independent and uncorrelated visits at the same designated GPS location.
- Function fitting precision: dispersion of stalk (mass density), $\rho_{S}$ and leaf biomass per cane, *BM*_*L*_, fitted functions, approximated by the overall root-mean-square-error (RMSE) based on the measured data and estimations from these fitted functions.

The instrument operation precision is part of the idealization precision and hence the two cannot be treated as independent. The idealization precision can also be interpreted as a small scale local variability. For both types, the measurements were taken five times consecutively and conducted once at early state (until 150 days of growth), once at mid state (between 150 and 300 days of growth) and once at late state (more than 300 days of growth). It is assumed that the measurements at these single dates are representative for the corresponding growth states. In case of cane spatial density, the instrument operation precision is the dispersion of the number of canes when counting the same part of the row five times. For cane spatial density idealization precision, neighboring rows were counted. The counting was carried out over five meters, typically consisting of 60 to 100 canes, and subsequently converted the cane density per meter. The instrument operation precision for leaf biomass per cane is approximated by the average of the s.d.’s from repetitive weight measurements of cane biomass, and assumed to be constant over growth states. The idealization precision of leaf biomass per cane is approximated by the average of s.d.’s of the cane biomass measured per ESUB, per growth state, scaled by the ratio between leaf biomass and cane biomass. Hence, for both cases, the assumption is made that variability in cane biomass is representative for variability in leaf biomass. For stalk (mass) density, the instrument operation precision and idealization precision are computed through error propagation of the biomass equation (Equation 1), resulting in:

(4)σΡS=ΡS(σBMS/BMS)2+(2σD/D)2+(σH/H)2

Here, $\sigma_{\rho_{S}}$, $\sigma_{BM_{S}}$, $\sigma_{D}$ and $\sigma_{H}$ are the precisions of stalk (mass) density ($\rho_{S}$), stalk biomass (*BM*_*S*_), cane diameter (*D*) and cane height (*H*), respectively. The ratio $\sigma_{BM_{S}}/BM_{S}$ is approximated by the equivalent of cane biomass, because at the ESUB locations only the variation in cane biomass was measured (see the subsec:Biomass section). The precisions of the parameters used in the biomass equation and LAI are shown in Table 2, expressed as one s.d. and valid for single observations. The idealization precision for cane spatial density at late stage is approximated by following the trend of precisions during the previous stages.

The function fitting precisions of stalk (mass) density and leaf biomass are not completely independent from the idealization precisions, because the functions are fitted through the measured parameters that are affected by the idealization precision (and consequently also by the instrument operation precision). The function fitting precisions of these parameters are shown in Table 3.

### Relative precisions of cane biomass and cane spatial density

Since the precisions of cane biomass are not directly measured, we apply error propagation laws on Equation 1, resulting in:

(5)σBMC=(ϑBMS+χBMS⋅|BMS|)2+(σBML)2

(6)ϑBMS=(2σDD)2+(σHH)2+(σρSρS)2χBMS=(σDD)2+(2σDσHDH)2+(2σDσρSDρS)2+(σHσρSHρS)2

Here, $\sigma_{BM_{C}}$, $\sigma_{D}$, $\sigma_{H}$, $\sigma_{\rho_{S}}$ and $\sigma_{BM_{L}}$ are the precisions associated to cane biomass (*BM*_*C*_), cane diameter (*D*), cane height (*H*), stalk (mass) density ($\rho_{S}$) and biomass leaf per cane (*BM*_*L*_), respectively, whereby Equation 4 is used for $\sigma_{\rho_{S}}/\rho_{S}$ and $\chi_{BM_{S}}$ is the second order term for biomass stalk per cane (*BM*_*S*_). For this error propagation, given the limited measurement data availability, it is assumed that the errors of the variables are uncorrelated and normal distributed. These equations have been applied with the instrument operation and idealization precisions from Table 2 for computing the corresponding precisions of cane biomass, $\sigma_{BM_{C}}$. These are expressed as percentages relative to the average *BM*_*C*_ estimates and shown in Table 4 for the corresponding stages. It shows that the instrument operation precision, as may be expected, is always smaller than the idealization precision.

Also incorporated in Table 4 is the function fitting precision that is a combination of the function fitting precisions of cane (mass) density and leaf biomass, both individually approximated by their function's RMSE (see Table 3). Since the absolute function fitting precision is taken as constant over time, the relative precision (expressed as percentages) decreases over increasing cane biomass. From the idealization precisions of *D* and *H* and the function fitting precisions of $\rho_{S}$ and *BM*_*L*_, the total precision at location is computed. The idealization precisions of $\rho_{S}$ and *BM*_*L*_ are not taken into account for the total precision at location, because these are not independent from the corresponding function fitting precisions. Consequently, the total precision at location is not a simple summation of these two precisions and results in smaller values than the idealization precisions for mid and late state. The difference between idealization precision and total precision can hence be interpreted as the effect of applying function fitting of $\rho_{S}$ and *BM*_*L*_.

In addition, from the measurements taken at each corner point of the ESUs and ESUBs, the ESU precision per stage is computed and shown in Table 3. These precisions are based on the averaged s.d. and the function fitting precisions, per stage. The table shows that the estimated variability within five meters (i.e. total precision at location) is similar yet slightly smaller than the variability within the ESU dimension of 20 by 20 meters (i.e. total precision within ESU).

The total precisions at location of Table 3 are used for perturbing the logistic fits of cane biomass of each field, as shown in Fig. 9. Explained in more detail, the uncertainty bars in Fig. 9, represented as one s.d. on either side of the logistic fit, were computed by taking the s.d. of hundred individual re-fitted logistic functions fitted through the measurements that were perturbed (normal) randomly based on the total precisions at location as input s.d.’s.

The same logic of precision estimation is applied to cane spatial density precisions ($\sigma_{C}$), see Table 5, again expressed as percentages relative to average cane spatial density. Fitting precisions are absent since no estimated parameters are involved. Although the absolute instrument operation precisions (i.e. counting error) decrease over the stages, see Table 2, the corresponding relative precision remains constant because of a similar decrease in cane spatial density. The relative idealization precisions decrease significantly over the stages, demonstrating the decrease in local variability. The ESU-related precisions are smaller than the location-related precisions, which could be explained by the apparent bias of the observer towards selecting and counting more dense sugarcane rows rather than randomly selecting sugarcane rows. Nevertheless, to remain consistent with the cane biomass case, for perturbing the logistic fits of cane spatial density again the total precisions at location were used. This is explained in more detail in the next section.

### Relative precisions of TCH

As briefly explained before, the estimated average tons cane per hectare (TCH) profiles of Fig. 10 are based on the multiplication of the logistic fits of cane biomass of each field with the logistic fits of cane spatial density of each ESU. For estimating the uncertainty of these profiles for each ESU, the field-dependent perturbed cane biomass profile is multiplied with the ESU-dependent perturbed cane spatial density profile. Both the perturbation of cane biomass measurements as well as the perturbation of cane spatial density measurements were carried out one hundred times, which means that the resulting uncertainty is based on ten thousand combinations of perturbed profiles. The uncertainty bars in Fig. 10 consequently represent the s.d. of the perturbed profiles on each side of the mean profile. Table 6 shows these precisions as percentages of the TCH per state averaged over all ESUs per field.

These relative TCH precisions show a significant reduction over time due to the increased TCH values over time and due to fitting of the logistic function for *BM*_*C*_ and *C* based on the perturbed measurements. This yields a significant difference compared to what would be expected when directly propagating the cane biomass precisions of Table 4 and the cane spatial precisions of Table 5. To quantify this difference (i.e. between the precisions associated to TCH based on the fitted functions and the precisions when TCH would be directly computed through the measurements), we the apply error propagation based on Equation 1 in combination with Equation 3, resulting in:

(7)σTCH=[(ϑTSH+χTSH⋅|TSH|︸1)2+(ϑTLH+χTLH⋅|TLH|︸2)2(ϑTSH+χTSH⋅|TSH|︸1)21/2

(8)ϑTSH=(2σDD)2+(σHH)2+(σΡSΡS)2+(σCC)2ϑTLH=(σBMLBML)2+(σCC)2χTSH=((σD)2D2)2+(2σDσHDH)2+(2σDσΡSDΡS)2+(2σDσCDC)2+(σHσΡSHΡS)2+(σHσCHC)2+(σΡSσCΡSC)2χTLH=(σBMLσCBMLC)2

In addition to the explained variables of Equation 5 and Equation 6, $\sigma_{TCH}$ and $\sigma_{C}$ are the precisions associated to tons cane per hectare (TCH) and cane spatial density (*C*), respectively, and $\chi_{TSH}$ and $\chi_{TLH}$ are the second order terms for tons stalk per hectare (TSH) and tons leaf per hectare (TLH). Similarly to Equation 5, the same error propagation assumptions apply and the results of Table 7 are expressed in percentages of the precisions relative to the average TCH estimates for the corresponding state. The precisions associated with the TSH and TLH are grouped separately (labeled group 1 and group 2, respectively) in order to show their magnitude relative to the TCH estimate at that state. The function fitting precisions again are approximated by the overall RMSE and in this case are based on the measured data and the fitted functions of cane spatial density, $\rho_{S}$, for group 1 and leaf biomass, $BM_{L}$, for group 2. Similarly to before, for these modeled parameters, only the fitting precisions are taken into account and their corresponding idealization precisions are disregarded. Also, the ESU-related precisions are again based on the averaged s.d. and the function fitting precisions, per stage. For group 1+2, the idealization precision (averaged s.d. without $\rho_{S}$ and $BM_{L}$ function fitting precisions) is included as well. This is used for comparison with the variogram at the end of this section.

The overall interpretation is that the leaf precision component, group 2, contributes less to the total cane precision, group 1+2, than the stalk precision component, group 1, at mid and late stage, which is logical given the overall weight dominance of stalk biomass to the cane biomass. The early stage leaf precision component is indeed dominated by the function fitting precision, due to the earlier mentioned relatively large fitting error and large weight portion of the plant during this state. This group also shows that in the early state the precisions are always larger than the mid and late states, which is due to the fact that the relative contribution of leaf biomass to the cane biomass decreases over time. Also, similar to before and as expected, the instrument operation precisions are always lower than the idealization precisions and the main contributor to the latter is the local variability in plant parameters. In case of group 2, the effect of local variability is apparently almost entirely dominating the idealization precision. The fact that the total precisions at location of group 2 is equal to the propagated sum of the idealization precisions and function fitting precisions shows that the contribution of leaf biomass to the idealization precision is small compared to the cane spatial density. This confirms the finding in the previous section that the contribution of cane spatial density to the total precisions is dominant.

A more detailed assessment of the results reveals that the instrument operation precisions of group 1 shows lower values for mid state than for the other stages, which is considered to be in the expected margin of error. In other words, it could be assumed these precisions should be viewed as constant over the stages. The total precision within ESU shows that these are comparable though slightly smaller (again assumed to be within the expected margin of error) than the total precisions at location, which means that the variability within an ESU is similar to the variability at the location. For all second order terms of the previous equations it holds that they only marginally contribute, up to 2% point, to the corresponding precisions, indicating that all higher order terms can be safely ignored.

The total precisions at location of Table 7 (based on error propagation) are generally larger than the precisions of Table 6 (based on profile perturbations) and there is a clear difference in temporal development of the precisions. For example, when TCH would be directly estimated from the measurements through Equation 3 and Equation 1, the precision is up to 45% of the TCH value (Table 7). This is significantly less precise than when the logistic fitting method of cane biomass and cane spatial density is used, where the precision is on average 18% in early state to 7% in late state of the TCH value (Table 6). This significantly lower precision is a result from the assumption that the boundary conditions of the general cane biomass growth curve can be used to constrain the field-dependent cane biomass growth curves in combination with the assumption both these curves and the ESU-dependent cane spatial density curves can be modeled by logistic functions. The advantage of this approach is that at any point in time (e.g. at the time of remote sensing images) in the growth period the TCH can be acquired, whereas with the direct measurements, interpolation and extrapolation needs to be carried out which induces another uncertainty. Hence, the modeled TCH curves per ESU are recommended for further use, e.g. for compare with remote sensing patterns in time and space.

**Figure 11:**
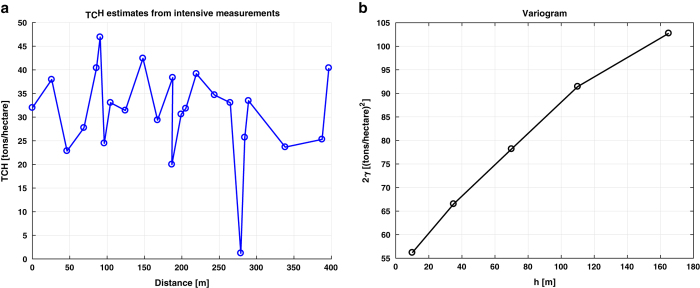
Biomass estimates from intensive measurements. (**a**) biomass estimates in TCH versus distance from intensive measurements at field F2; (**b**) corresponding variogram, where the variance (2*γ*) in (TCH)2 is function of the lag distance *h*.

The final set of measurements for describing the TCH variability was taken on a single date, 19 December 2014 (66 days after start of growth), in field F2. These were taken along a profile, starting from point F2-IM1 in Fig. 4 until 400 meters north from that point, every 5, 10 and 20 meters along that line, resulting in 24 local measurement locations. At every location, leaf length, stalk height, stalk thickness, cane spatial density, plant spatial density, LAI and soil moisture were measured. At each location, biomass values were computed from the biomass re-fitted logfit functions per field of Fig. 9 multiplied by the measured cane spatial densities. The results are shown as a profile along the measurement line and as a variogram, see Fig. 11. The variance (2*γ* as function of lag distance *h*) in each bin was estimated from 36 to 61 sample pairs. The associated s.d. as percentage of the averaged estimated biomass (approximately 30 tons/ha) is 24% within the spatial dimension of an ESU (*h*=20 meters) and ranges to nearly 33% at the maximum calculated distance (*h*=160 meters). The former value is lower than the early state idealization precision of group 1+2 but similar to the idealization precision within ESU of group 1+2 in Table 7.

## Usage Notes

In (Data Citation 1), the readme files explains the location of the files and folders. All raw measurements records can be found in one Excel sheet, which is subsequently used in MATLAB for further processing and analysis.

## Additional information

**How to cite this article**: Molijn, R. A. *et al*. Ground reference data for sugarcane biomass estimation in São Paulo state, Brazil. *Sci. Data* 5:180150 doi: 10.1038/sdata.2018.150 (2018).

**Publisher’s note**: Springer Nature remains neutral with regard to jurisdictional claims in published maps and institutional affiliations.

## Supplementary Material



## Figures and Tables

**Table 1:** Sugarcane fields’ characteristics with growth and harvest dates, which are for F1 and F2 not applicable to the entire field.

**Field name**	**Ratoon**	**Area [ha]**	**Start of growth**	**Harvest**	**Center coordinate [latitude, longitude]**
F1	1st cycle	58	30/10/2014	07/10/2015	−22.9607, -47.4578
F2	1st cycle	115	14/10/2014	07/12/2015	−22.8558, -47.4228
F3	2nd cycle	25	15/08/2014	26/07/2015	−22.8738, -47.5230
F4	9th cycle	59	01/08/2014	21/07/2015	−22.8895, −47.5108

**Table 2:** Absolute instrument operation and idealization precisions, mainly based on the three intensive measurement campaigns in three growth stages.

	**Instrument operation**			**Idealization**		
Parameter	Early	Mid	Late	Early	Mid	Late
Stalk thickness [cm]	0.2	0.2	0.3	0.2	0.3	0.4
Stalk height [cm]	0.8	10.2	11.8	5.6	32.9	25.9
Cane spatial density [stalks/row/m]	0.8	0.7	0.5	6.3	4.0	2.1
LAI [m^2^/m^2^]	0.2	0.2	0.3	0.2	0.4	0.4
Stalk (mass) density [kg/m^3^]	73	60	84	84	121	143
Leaf biomass per cane [g]	6.1	6.1	6.1	14.6	31.2	15.3

**Table 3:** Absolute fitting precisions, approximated by the RMSE of the fitted functions, which are based on the measurements in the ESUBs.

**Parameter**	**Fitting precision**
Stalk (mass) density [kg/m^3^]	65
Leaf biomass per cane [g]	58
It is assumed these are constant over the growth states.	

**Table 4:** Relative precisions with respect to cane biomass (*BM*_*C*_).

**Cane biomass** **(*****BM***_***C***_**)**	**Early state**	**Mid state**	**Late state**
Idealization precision	21%	30%	32%
(Instrument operation precision)	(19%)	(16%)	(20%)
Function fitting precision	29%	14%	12%
Total precision at location	32%	23%	23%
Total precision within ESU	36%	24%	27%

**Table 5:** Relative precisions with respect to cane spatial density (*C*).

**Cane spatial density (*****C*****)**	**Early state**	**Mid state**	**Late state**
Idealization precision	45%	29%	19%
(Instrument operation precision)	(5%)	(5%)	(5%)
Total precision at location	45%	29%	19%
Total precision within ESU	23%	16%	10%

**Table 6:** Relative precisions with respect to TCH as a result from perturbation of the measurements in the logistic fits. The results per ESUs are averaged per field.

**Field**	**Early state**	**Mid state**	**Late state**
F1	13%	9%	9%
F2	9%	8%	7%
F3	19%	7%	6%
F4	31%	7%	7%
Average	18%	8%	7%

**Table 7:** Relative precisions with respect to tons cane per hectare (TCH) as a result from error propagation from repeated measurements.

**Group 1: stalk component (TSH)**	**Early state**	**Mid state**	**Late state**
Idealization precision (Instrument operation precision)	31% (19%)	38% (17%)	36% (21%)
Function fitting precision	12%	12%	11%
Total precision at location	30%	32%	29%
Total precision within ESU	27%	27%	28%
Group 2: leaf component (TLH)	Early state	Mid state	Late state
Idealization precision (Instrument operation precision)	22% (4%)	6% (1%)	2% (1%)
Function fitting precision	26%	6%	5%
Total precision at location	34%	8%	5%
Total precision within ESU	28%	6%	5%
Group 1+2: total cane (TCH)	Early state	Mid state	Late state
Idealization precision (Instrument operation precision)	38% (19%)	38% (14%)	36% (12%)
Function fitting precision	29%	14%	12%
Total precision at location	45%	33%	29%
Idealization precision within ESU	26%	24%	25%
Total precision within ESU	39%	27%	28%
The results are given for the stalk and leaf components individually and for the total cane.			
