# Author Correction: Ground reference data for sugarcane biomass estimation in São Paulo state, Brazil

**DOI:** 10.1038/s41597-019-0309-x

**Published:** 2019-12-02

**Authors:** Ramses A. Molijn, Lorenzo Iannini, Jansle Vieira Rocha, Ramon F. Hanssen

**Affiliations:** 1 Geoscience and Remote Sensing, Delft University of Technology, Delft, 2628CN The Netherlands; 2 FEAGRI, Unicamp, Campinas, 13083-875 Brazil

Correction to: *Scientific Data* 10.1038/sdata.2018.150, published online 07 August 2018

Following publication, it was noticed that the horizontal brackets labelling the two groups of precisions present in Equation 7 are incorrectly rendered in the PDF version of this Data Descriptor. The correct Equation 7 is as follows:

$$\begin{array}{lll}{\sigma }_{TCH} & = & \left[{\left(\mathop{\underbrace{\sqrt{{\vartheta }_{TSH}+{\chi }_{TSH}}\cdot \left|TSH\right|}}\limits_{1}\right)}^{2}\right.+\\  &  & +\,{\left.{\left(\mathop{\underbrace{\sqrt{{\vartheta }_{TLH}+{\chi }_{TLH}}\cdot \left|TLH\right|}}\limits_{2}\right)}^{2}\right]}^{1/2}\end{array}$$

In addition, in the Biomass subsection of the Methods section in both the HTML and PDF versions, the term “ESUs” is incorrectly rendered as “ESUâ€™s” and the term ESUBs is incorrectly rendered as “ESUBâ€™s”

Finally, throughout the manuscript, references to sections and subsections include the prefixes “sec:” and “subsec:”, respectively. These prefixes and any hyphen between the reference words that follow the prefixes can be ignored.

