# Author Correction: Ground reference data for sugarcane biomass estimation in São Paulo state, Brazil

**DOI:** 10.1038/s41597-020-0525-4

**Published:** 2020-06-17

**Authors:** Ramses A. Molijn, Lorenzo Iannini, Jansle Vieira Rocha, Ramon F. Hanssen

**Affiliations:** 1 Geoscience and Remote Sensing, Delft University of Technology, Delft, 2628CN The Netherlands; 2 FEAGRI,Unicamp, Campinas, 13083-875 Brazil

Correction to: *Scientific Data* 10.1038/sdata.2018.150, published online 07 August 2018

Following publication, an erroneous element was found in Equation 1, where $\pi$ was incorrectly squared. The correct notation is:

$$B{M}_{C}=B{M}_{S}+B{M}_{L}=\left(\frac{\pi {D}^{2}}{4}\cdot H\cdot {\rho }_{S}\left(t\right)\cdot F\right)+B{M}_{L}\left(t\right)$$

In addition, the text “scaling factor C in the equation” in the Methods should instead read as “scaling factor F in the equation”, as the scaling factor in Equation 1 is denoted as “F” and not “C”.

The correct equation and notation were utilised in this study, hence neither correction affects the scientific results of the paper.

